# Quality not quantity: The value of qualitative research

**DOI:** 10.1111/1471-0528.17149

**Published:** 2022-04-03

**Authors:** Elaine Denny, Annalise Weckesser

**Affiliations:** ^1^ Faculty of Health, Education and Life Sciences Birmingham City University Birmingham UK; ^2^ Centre for Social Care and Health Related Research Faculty of Health, Education and Life Sciences Birmingham City University Birmingham UK

Learning points

- The findings from qualitative research (QR) have much to offer medical science.
- Findings from QR lead to an understanding of the perspectives of those experiencing the event under investigation.
- It may answer questions around ‘how’ and ‘why’, as well as ‘what’.

Qualitative research plays a valuable role in complementing quantitative research, although traditionally it has been undervalued within the medical sciences. Clinical trials, other experimental evaluations and cohort studies are usually prospective observations of large numbers. In contrast, qualitative research is more in‐depth, exploring small populations that share a common characteristic or attribute, often by suffering from a specific health problem (Bryman. *Social Research Methods*. Oxford: Oxford University Press, 3rd edn; 2008). For example, when embedded in pilot or feasibility studies, pertinent insights into the acceptability of an intervention may be elicited because participants can introduce and reflect upon issues of importance to them. Although not generalisable to the wider population, qualitative research may lead to knowledge that can improve patient experience and outcomes.

Qualitative research allows stakeholders to influence research by raising issues that have not have been considered. It can react to the unfolding of events and constant review may lead to the adaptation of research questions or interview guidelines. This is not necessarily a linear process, but may be cyclical. However, to increase rigour it is important that the process is transparent and traceable.

Findings from QR lead to an understanding of the social world from the standpoint of those experiencing the social phenomenon or event under investigation. It provides rich, in‐depth data, and can answer ‘how’ and ‘why’ questions, as well as ‘what’. QR explores complexities, nuance and seemingly contradictory positions that people may hold in the context of their lives, and the experience of people who are typically under‐represented in experimental studies, such as clinical trials.

The following study exemplifies the value of qualitative research. A mixed methods study, combining a quantitative survey with a qualitative interviewing, on experiences of women undergoing termination of pregnancy (TOP) compared those who had:

1. never undergone a TOP before,
2. one TOP more than 2 years ago,
3. more than one TOP within the previous 2 years.

The quantitative survey revealed that the third group were less likely to own their own homes, and more likely to report both consistent and inconsistent contraceptive use. Qualitative interviews were then conducted with 23 of the women from the third group. It was found that most women from this group were not using TOP as contraception, and had tried various methods to prevent pregnancy. It was also found that women from this group had specific vulnerabilities, including inter‐personal violence, mental health problems, socio‐economic disadvantage and relationship difficulties. Although the experiences and reasons reported by participants are familiar, the women described distinct experiences for each pregnancy, not just a repeating scenario, which highlights the dynamic and complex nature of women’s lives (Purcell et al. Women’s experiences of more than one termination of pregnancy within two years: a mixed methods study. *BJOG* 2017;**124**:1983–92). Contextualising the vulnerabilities highlighted provides an understanding of women’s needs around the time of a TOP that can influence the provision of sensitive healthcare.

## 1 RESOURCES

This open letter to the editors of the *BMJ* from 76 researchers provides a coherent argument for medical journals to be more receptive to the findings of qualitative research.

- http://www.bmj.com/content/bmj/352/bmj.i563.full.pdf

This website provides a comprehensive guide to designing, reporting and reviewing qualitative research in healthcare settings.

- http://www.qualres.org/

## CONFLICT OF INTERESTS

None declared. Completed disclosure of interest forms are available to view online as supporting information.

## AUTHOR CONTRIBUTIONS

Elaine Denny and Annalise Weckesser contributed equally to the paper.

## Supporting information


**Appendix** S1

**Appendix** S2

